# SimuSCoP: reliably simulate Illumina sequencing data based on position and context dependent profiles

**DOI:** 10.1186/s12859-020-03665-5

**Published:** 2020-07-23

**Authors:** Zhenhua Yu, Fang Du, Rongjun Ban, Yuanwei Zhang

**Affiliations:** 1 School of Information Engineering, Ningxia University, Yinchuan, 750021 China; 2 Hefei National Laboratory for Physical Sciences at Microscale, USTC-SJH Joint Center for Human Reproduction and Genetics, School of Life Sciences, University of Science and Technology of China, Hefei, 230027 China

**Keywords:** Next-generation sequencing, Simulators, Base substitution errors, Phred base quality, Intra-tumor heterogeneity

## Abstract

**Background:**

A number of simulators have been developed for emulating next-generation sequencing data by incorporating known errors such as base substitutions and indels. However, their practicality may be degraded by functional and runtime limitations. Particularly, the positional and genomic contextual information is not effectively utilized for reliably characterizing base substitution patterns, as well as the positional and contextual difference of Phred quality scores is not fully investigated. Thus, a more effective and efficient bioinformatics tool is sorely required.

**Results:**

Here, we introduce a novel tool, SimuSCoP, to reliably emulate complex DNA sequencing data. The base substitution patterns and the statistical behavior of quality scores in Illumina sequencing data are fully explored and integrated into the simulation model for reliably emulating datasets for different applications. In addition, an integrated and easy-to-use pipeline is employed in SimuSCoP to facilitate end-to-end simulation of complex samples, and high runtime efficiency is achieved by implementing the tool to run in multithreading with low memory consumption. These features enable SimuSCoP to gets substantial improvements in reliability, functionality, practicality and runtime efficiency. The tool is comprehensively evaluated in multiple aspects including consistency of profiles, simulation of genomic variations and complex tumor samples, and the results demonstrate the advantages of SimuSCoP over existing tools.

**Conclusions:**

SimuSCoP, a new bioinformatics tool is developed to learn informative profiles from real sequencing data and reliably mimic complex data by introducing various genomic variations. We believe that the presented work will catalyse new development of downstream bioinformatics methods for analyzing sequencing data.

## Background

As next-generating sequencing (NGS) techniques have become the current standard for profiling genomes, large amount of data is extensively accumulated in last decade, but the downstream analysis of these data remains a bottleneck [1]. Therefore, an arsenal of bioinformatics tools is constantly being complemented to provide improved processing ability and inference performance. Benchmarking the newly developed methods against existing tools is essential to examine their advantage in some specific aspects, and simulation of sequencing data has become a popular approach to provide baselines for comparison.

The inner complexity of NGS technologies gives rise to numerous challenges in reliably emulating sequencing data. It is well known that in NGS experiments base-calling errors often arise in the procedure of translating sensor signals to distinct nucleotides, and this type of errors is dominant in Illumina sequencing platforms [2]. Note that base substitution errors may also occur during PCR amplification of the DNA templates [3]. To measure the quality of each base call, Phred quality score is defined as a prediction of base-calling error probability that can be used to discriminate between correctly and erroneously called bases [4]. The existing studies demonstrate that specific patterns of substitution error and distributions of quality scores are observed in Illumina sequencing platforms [5, 6]. Fully investigating these statistical differences in NGS reads is essential to obtain useful knowledge that can be employed to improve the read alignment quality, and to emulate reliable sequencing data.

So far, numerous tools have been developed for simulating DNA sequencing data for specific applications (Table 1). These tools show distinct features in multiple aspects including read layout (single end, paired-end and mate pair), output format (FASTQ, SAM and FASTA), programming language, supported sequencing strategy (whole-genome sequencing or/and exome sequencing), error models (positional or/and contextual dependent), support for GC bias, genomic variation, and tumor sample simulation. The common pipeline to simulate sequencing data consists of two steps: 1) manipulate input reference sequence to generate genomes from which reads are to be captured; 2) sample reads from the produced genomes and insert sequencing errors into the reads under a deterministic or stochastic manner. The first step involves insertion of various genomic aberrations including single nucleotide variation (SNV), copy number variation (CNV), loss of heterozygosity (LOH), indel and other structure variations (SV) into the reference sequence [8, 9, 15, 16, 18, 19]. For instance, pIRS [9] randomly insert variations into the reference sequence under fixed frequency. Pysim-sv [19] can simulate both germline and somatic genomic variations. Specifically, it is capable of emulating heterogeneous subclones in an iterative way and generating tumor samples by mixing different cell populations. Similar to Pysim-sv, SCNVSim [16] mimics tumor subclones using an iterative manner. Another tool called IntSIM [18] uses hidden Markov models (HMM) to imitate germline or somatic variations, and can generate reads for impure tumor samples by mixing tumor and normal genomes. There are several tools that can simulate genomic variations provided that the baseline reference sequence is preprocessed to include the variations to be simulated, such as ART [7], InSilicoSeq [20] and FASTQSim [14]. This gives rise to inconvenience and challenge for non-professional users to accurately generate underlying genome sequences.

**Table 1:** A brief summary of existing tools for simulating DNA sequencing data

Simulator	Layout^**b**^	Output	Language	Genomic variation			Tumor sample			GC bias	Profiles		Sequencing strategy^**c**^	Ref
				SNV	CNV	Indel	Impurity	Aneuploidy	Intra-tumor heterogeneity		Position dependent	Context dependent		
ART	SE, PE	FQ, SAM	C++, Perl								X		G	[7]
Grinder	SE, PE	FQ, FA	Perl		X						X		G	[8]
pIRS	PE	FQ	C++, Perl	X	X	X				X	X		G	[9]
GemSIM	SE, PE	FQ, SAM	Python	X							X	X	G	[10]
Wessim^a^	SE, PE	FQ, SAM	Python							X	X	X	E	[11]
NeSSM	SE, PE	FQ	C, Perl							X	X		G	[12]
BEAR	SE, PE	FQ	Perl, Python								X		G	[13]
FASTQSim	SE	FQ	Python								X		G	[14]
SInC	PE	FQ	C	X	X	X					X		G	[15]
SCNVSim^a^	SE, PE	FQ	Java	X	X	X	X	X	X		X		G	[16]
NEAT	SE, PE	FQ	Python	X	X	X				X	X		G, E	[17]
IntSIM	SE, PE	FQ	C++, Perl, R	X	X	X	X	X	X	X	X		G	[18]
Pysim-sv^a^	SE, PE	FQ	Python	X	X	X	X	X	X	X	X		G	[19]
InSilicoSeq	PE	FQ	Python							X	X		G	[20]
SimuSCoP	SE, PE	FQ	C++	X	X	X	X	X	X	X	X	X	G, E	

X: a given functional capability is supported by a simulator. ^a^: these tools depend on third party NGS read simulator to generate reads. ^b^: SE denotes single end and PE represents paired-end. ^c^: G denotes whole-genome sequencing, and E indicates target or exome sequencing

In the second step of the read simulation, base sequences are randomly copied from the genomes, and further processed to introduce sequencing errors including base substitutions and indels based on specific error models. The length of reads can be fixed or sampled from a probability distribution [13]. The error models are used to describe statistical distributions of base substitution errors, indels and quality scores, which can be learned from real sequencing data. There are two types of error models implemented in current tools, defined as position and context dependent models. The position dependent model captures the relationship between sequencing errors and base positions, and is usually represented by per-position probability distributions of the errors. The existing simulators almost invariably build this type of models and conclude some meaningful perspectives: the base substitution error tends to happen at a much higher rate near the end of read, and the larger the base position, the higher the error rate. For instance, ART models substitution errors to be positional dependent, and profiles indel errors from real training datasets. pIRS generates base sequence according to a distribution matrix that stores the base-calling information in all read cycles derived from real sequencing data, and yields quality scores based on a quality-transition matrix representing the correlation between adjacent bases. Similar model is adopted by BEAR [13] to generate base quality for correct base calls, and a second-degree polynomial regression is used to sample quality values for erroneous base calls. More recently, InSilicoSeq is introduced to accurately model per-base quality scores using Kernel Density Estimation [21], and able to reliably produce reads that show highly consistent base quality distributions with the underlying truth.

It is noteworthy that existing studies report that substitution errors are also closely correlated with the genomic sequence contexts and show specific patterns for different sequencing platforms [22, 23]. This kind of features cannot be fully covered by the positional information, therefore context dependent error models should be also built to enable the comprehensive profiling of sequencing errors. To our knowledge, GemSIM [10] is the only one method that supports both position and context dependent models. It tracks three bases preceding each position of the read and corresponding quality scores to simulate substitution errors. Deep mining of the contextual information is still highly needed to strength our knowledge about the underlying substitution patterns and provide aid to more accurate simulation of data.

GC-content bias is another factor that should be considered when generating reads. The existing studies have demonstrated GC-content is one of the main factors that affect depth of coverage (DOC) of a genomic region, and leads to non-uniform distribution of reads along the genome [24, 25]. Building the relationship between DOC and GC-content using appropriate models is essential to emulate GC-content bias presented in real data, and guide the development of effective methods for alleviating GC-content bias. Several simulators have explicitly modeled this bias [9, 11, 12, 17–19, 26]. For instance, pIRS models GC-content bias by sampling a read with the probability proportional to the mean coverage associated with the GC-content of the read. IntSIM does not use the coverage information and employs a linear function of GC-content to denote the probability of generating a read. Wessim [11] is specifically designed to emulate whole-exome sequencing data, and employs a probability partially defined by GC-content to filter generated fragments.

Despite the high efficiency achieved by current NGS simulators, their practicality may be degraded by functional and runtime limitations. For instance, both pIRS and Pysim-sv provide no options for users to specify the exact locations of the simulated variations. Although a delicate representation of read simulation process is introduced in IntSIM, it is memory inefficient when inferring quality profiles from raw sequencing data. InSilicoSeq can only generate reads with fixed length estimated from a BAM file, and is of memory inefficiency when simulating a large amount of reads. Particularly, the substitution patterns based on contextual information are not fully investigated in the existing simulators.

Here we present a novel bioinformatics tool called SimuSCoP to complement the arsenal of tools for emulating complex DNA sequencing data. Compared to existing tools, SimuSCoP gets substantial improvements in reliability, functionality, practicality and runtime efficiency. First, the base substitution patterns and the statistical behavior of quality scores in Illumina sequencing data are fully explored from both positional and contextual views to simulate more reliable reads. Second, effective implementations of biological (indel, SNV, CNV and tumor heterogeneity) and technological features (whole-genome or exome sequencing, read layout) enable SimuSCoP to meet requirements of different applications. Third, an integrated and easy-to-use pipeline is employed in SimuSCoP to facilitate end-to-end simulation of complex samples. Finally, high runtime efficiency is achieved by implementing the tool to run in multithreading with low memory consumption. We comprehensively evaluate the tool from multiple aspects, and the results demonstrate SimuSCoP’ advantages over existing tools.

## Implementation

**Fig. 1:**
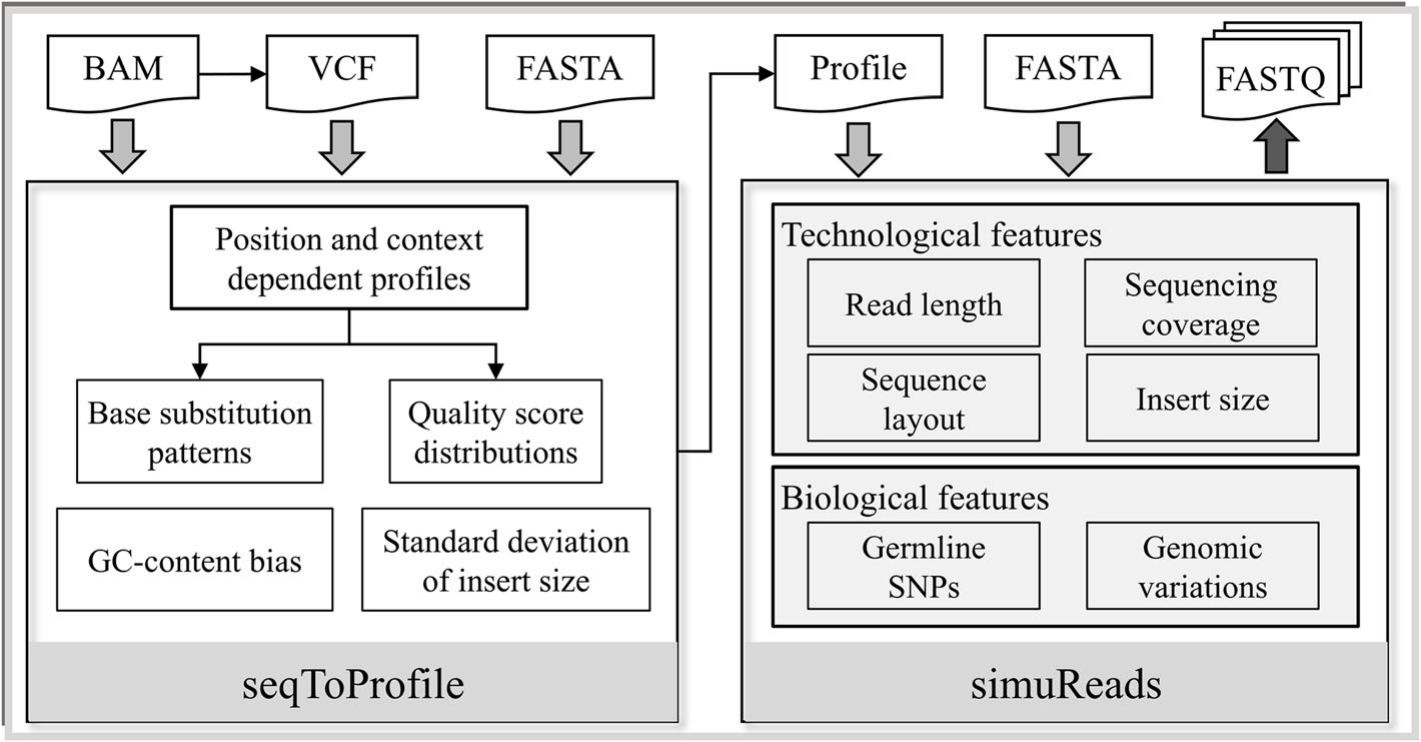
Overview of the SimuSCoP framework. Two functional modules are implemented to learn profiles from real data and simulate reads

SimuSCoP consists of two modules as shown in Fig. 1: 1) inference of base substitution patterns, base quality distributions, GC-content bias and standard deviation of insert size from aligned NGS reads; and 2) simulation of complex NGS data using the learned profiles. The first module takes three inputs: 1) a BAM file of non-tumor sample; 2) a FASTA file of the reference sequence to which the reads are aligned; and 3) a VCF file generated from the BAM using GATK [27] HaplotypeCaller utility or SAMtools [28]. For exome sequencing, a BED file defining target regions should also be provided. The second module combines the inferred profiles, user-defined technological features and various genomic variations to yield complex NGS data.

### Profile inference

#### Inference of base substitution patterns

For estimating base substitution patterns, the alignments with high mapping quality (> 15) from the BAM file as well as the data in the FASTA and VCF files are extracted to construct all pairs of read sequence and corresponding source sequence. The source sequence is the underlying sequence from which the read is generated. The VCF file defines the germline heterozygous SNPs inferred from the BAM file, and is used to eliminate non-error substitutions. To characterize the difference of substitution patterns between distinct positions within the read, we divide all positions into equal-sized bins and infer substitution patterns for each bin separately. Using the source sequence *X* as the baseline for comparison, each base *Y*_*i*_ of read sequence *Y* is evaluated to measure the probabilities being substituted for other base y ϵ ${\Delta}_{Y_i}$, here ${\Delta}_{Y_i}$ is the set of all bases (A, C, T and G) except *Y*_*i*_. On the other hand, to explore the effects of genomic contexts on base substitution errors, the base of each position *i* is considered to be dependent on the *k*-mer bases (*X*_*i-k + 1*_,*X*_*i-k + 2*_, *…*,*X*_*i*_) derived from the source sequence, and the conditional probability of *Y* is defined as follows:

1 $$ p\left(Y\left|X\right.\right)=\prod \limits_{i-1}^{k-1}p\left({Y}_i\left|{X}_1,{X}_2,\mathrm{K},{\mathrm{X}}_i\right.\right)\prod \limits_{i=k}^Tp\left({Y}_i\left|{X}_{i-k+1}\right.,{X}_{i-k+2},\mathrm{K},{X}_i\right) $$

where *T* is the length of read sequence. The probability model in (1) effectively characterizes the dependency of observed read on both positional and contextual information contained in the source sequence. We estimate each item in (1) as the occurrence frequency of corresponding substitution given the *k*-mer bases, and the profiles are separately inferred for forward and reverse reads in paired-end sequencing.

#### Inference of base quality distributions

To examine the difference of quality scores among different positions within the read, the statistical distributions of quality scores of each nucleotide are evaluated. Similarly, the positions are also divided into equal-sized bins, and the statistics are separately measured for each bin. Suppose the quality scores of the readout nucleotide sequence are denoted by *Z*, the conditional probability of *Z* is defined as:

2 $$ p\left(Z\left|X,Y\right.\right)=\prod \limits_{i=1}^Tp\left({Z}_i\left|{X}_i,{Y}_i\right.\right) $$

where we assume the quality scores of different positions are independent, and the quality score of each base position only depends on the bases at the corresponding position. *p* (*Z*_*i*_|*X*_*i*_,*Y*_*i*_) represents the score probability of the *i*-th base and depends on the positional and contextual information. By comparing the bases at the same positions within the read sequence *Y* and source sequence *X*, the status of the base pair (*X*_*i*_,*Y*_*i*_) can be represented by one of the following scenarios: 1) *X*_*i*_ is correctly called (*X*_*i*_ = *Y*_*i*_); 2) *X*_*i*_ is erroneously called (*X*_*i*_ ≠ *Y*_*i*_). Therefore we define the *p* (*Z*_*i*_|*X*_*i*_,*Y*_*i*_) as:

3 $$ p\left({Z}_i\left|{X}_i,{Y}_i\right.\right)={p}_c\left({Z}_i\left|{Y}_i\right.\right){\mathrm{I}}_{X_i={Y}_i}+{p}_e\left({Z}_i\left|{Y}_i\right.\right)\left(1-{\mathrm{I}}_{X_i={Y}_i}\right) $$

where *p*_*c*_ (*Z*_*i*_|*Y*_*i*_) and *p*_*e*_(*Z*_*i*_|*Y*_*i*_) are the respective quality probabilities under conditions *X*_*i*_ = *Y*_*i*_ and *X*_*i*_ ≠ *Y*_*i*_, and ${\mathrm{I}}_{X_i={Y}_i}$ is an indicator function. We calculate the respective occurrence frequency of quality score *Z*_*i*_ as the probability *p*_*c*_ (*Z*_*i*_|*Y*_*i*_) and *p*_*e*_ (*Z*_*i*_|*Y*_*i*_).

#### Inference of GC-content bias

To explicitly describe the effects of GC-content on depth of coverage (DOC), normal distributions are used to represent the distributions of the DOC corresponding to different GC percentages. For whole-genome sequencing (WGS) data, the DOC and GC percentage of non-overlapping 1 kb windows are obtained. For target sequencing, the DOC and GC percentage of each target region are measured, and the DOC data is further normalized for target size. Whereafter, median normalization is applied to the DOC data, and the mean values associated with each GC percentage are inferred by adopting locally weighted linear regression of the DOC over GC percentage. The standard derivation of DOC is then calculated as:

4 $$ {\sigma}_d=\sqrt{\sum \limits_{i=1}^N{\left({d}_i-{m}_i\right)}^2/N} $$

where *d*_*i*_ is the DOC of the *i*-th window, *m*_*i*_ is the DOC mean value, and *N* is the number of windows.

### Simulation of reads

#### Simulating single read

To simulate a read from a given source sequence, indels of different lengths are first randomly inserted into the source sequence under fixed occurrence rate. The indel error rate and the distributions of indel length are inferred from real samples, and the bases of insertions are randomly sampled from the nucleotides “ACGT”. The read sequence is then generated by sampling nucleotides from the conditional probability distributions given the source sequence. The quality scores are produced under two different scenarios: for correctly called nucleotides, the quality scores are sampled from the probability distribution *p*_*c*_(z|*y*); for erroneously called nucleotides, *p*_*e*_(*z*|*y*) is used to generate quality scores. This process will yield a sequence pair (*Y*, *Z*) from source sequence *X*.

#### Simulating reads from a genomic region

To sample *M* reads from a genomic region, the region is first divided into non-overlapping 1 kb windows, and the normalized DOC of each window is sampled from the normal distribution *N*(*m*_*g*_, *σ*_*d*_), here *g* is the GC percentage of the window and *m*_*g*_ is the mean DOC associated with GC *g*. The DOC data *D* is further normalized to calculate the number of reads sampled from the *i*-th window as *M*_*i*_ = *D*_*i*_/∑_*j*_*D*_*j*_. For single end sequencing, fixed-length fragments are randomly obtained from each window and base sequence is captured from either ends of each fragment. For paired-end sequencing, fragments of normal distributed length are first sampled from each window, and two base sequences are generated from the ends of each derived fragment. The produced base sequences are used as source sequences to generate reads by using aforementioned approach. This process will produce *M* sequence pairs from a genomic region.

#### Simulating reads from mixed genomes

To sample *N* reads from heterogeneous samples mixed by multiple distinct genomes or cell populations, the average copy number (ACN) of each genome is first calculated. The number of reads sampled from each genome is then empirically measured as ${N}_i={w}_i{P}_iN/\sum \limits_{j=1}^G{w}_j{P}_j$. Here we use *w*_*i*_ and *P*_*i*_ to denote the proportion and ACN of the *i*-th genome respectively, and *G* to represent the number of mixed genomes in the heterogeneous sample. Reads are emulated from each genome by using the approach described in the previous section “*Simulating reads from a genomic region*”.

### Simulation of complex data

In the second module of SimuSCoP, a configuration file is used to specify the all aspects of the sequencing data. As shown in Fig. 1, the main parameters and profiles include: 1) a FASTA file of the reference sequence from which reads are to be generated; 2) the profiles inferred by the first module of SimuSCoP; 3) germline SNPs and genomic variations to be simulated; and 4) technological features including sequence layout (single end, paired end), read length, sequencing coverage and insert size (for paired end sequencing). The following sections give a detailed description of the configurations.

#### Simulating SNPs

The required fields to specify each SNP include the name, chromosome, position, observed nucleotides, strand, and reference allele of the SNP. The commonly used SNP data can be download from https://genome.ucsc.edu/cgi-bin/hgTables. Here we only consider biallelic SNPs when generating the genome sequences. For instance, to construct a diploid genome, we first use two replicates of the reference sequence as templates, then iteratively insert the wild allele of each SNP into one of the template sequences. We employ the produced genome as a baseline to generate other genomes by inserting various genomic variations.

#### Simulating genomic variations

The simulated genomic variations such as SNVs, CNVs and indels can be germline or somatic. The required information to define different variations is explained as follows: 1) A SNV is specified by the aspects including the name of the chromosome, the position of the mutation, reference allele, mutated allele and the type of the mutation (homozygous or heterozygous); 2) a CNV is depicted by the fields including the chromosome, start position, end position, total copy number, and major allele copy number; 3) a short insert is defined by the chromosome, insert position and the nucleotide sequence to be inserted; and 4) a deletion is represented by the chromosome, start position and the length of the deletion. For homozygous SNV, the alleles at corresponding position from both template sequences are set to mutated allele, and for heterozygous SNV, the allele of a randomly selected template sequence is modified to the mutated allele of the SNV. To simulate indels, the template sequences are manipulated by inserting nucleotide sequence into specific position or erasing predefined length of bases from the templates. Whereafter, CNV is emulated by duplicating the specified genomic region of one template sequence *m* times, and duplicating the region of another template (*n*-*m*) times, here *n* is the total copy number and *m* is the major allele copy number of the CNV. Following the presented procedures, we can generate the underlying genomes ready for sequencing.

#### Simulating tumor samples

Tumor sample is often complicated by issues of impurity, aneuploidy and intra-tumor heterogeneity. To reliably emulate tumor samples, multiple tumor genomes corresponding to different clones are generated and mixed with the baseline genome (the genome of normal cell) at given proportions. The names indicating each component of the mixed genomes and an abundance file providing the mixing proportions are defined in the configuration file. The sequencing data of the tumor sample is obtained by using the aforementioned approach for simulating reads from mixed genomes.

## Results

### Real sequencing data

To investigate the profiles of the samples generated from different sequencing platforms, the FASTQ files of 8 samples (Table S1 in Additional file 1) are downloaded from the Sequence Read Archive (SRA) of NCBI by using SRA ToolKit. These samples are assayed by Illumina Genome Analyzer IIx, HiSeq 2000, HiSeq 2500 or HiSeq X 10 instrument. The reads are aligned to the hg19 human reference genome using BWA [29] tool, and germline SNPs are further inferred from the BAM files by using GATK HaplotypeCaller under default parameters.

### Base substitution patterns

**Fig. 2:**
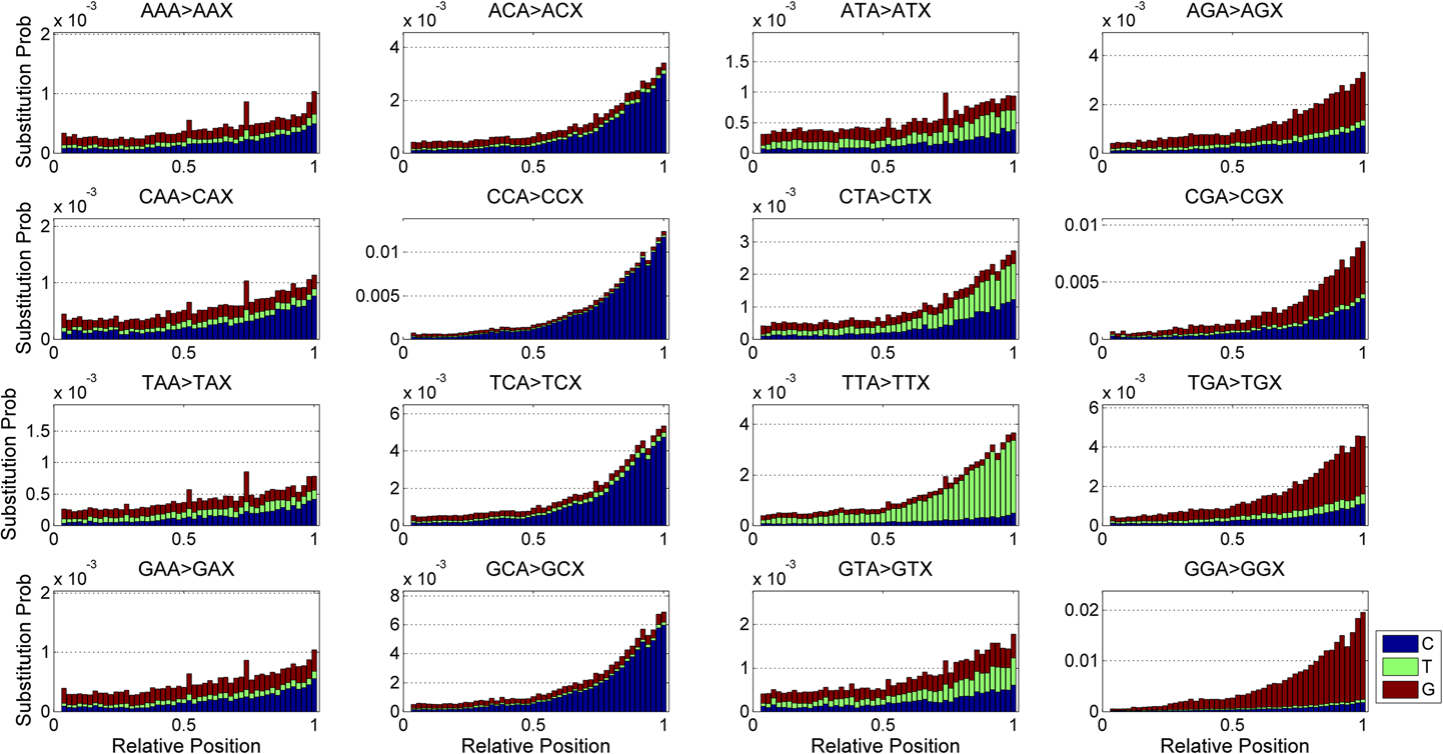
Base substitution probabilities of nucleotide A inferred from forward reads of sample SRR1614306. The conditional occurrence frequency of each base substitution under 3-mer bases derived from source sequence is measured

We analyze the base substitution patterns in forward reads of sample SRR1614306. Figure 2 shows the conditional probabilities of substituting nucleotide A for other nucleotides under the 3-mer bases derived from the source sequence. Same to the previously reported results [10, 18], the overall error rate generally increases towards to the end of the reads. More significantly, the substitution patterns are different among distinct nucleotides. The conversions (XCA > XCC), (TTA > TTT), (TGA > TGG) and (GGA > GGG) are dominant when the relative base position is larger than 0.5, here X denotes any nucleotide. Particularly, (CCA > CCC) and (GGA > GGG) consistently represent the most significant error types among all substitutions, which indicates a strong tendency of substituting A for C and G when the preceding bases are (CC) and (GG) respectively.

We then explore the base substitution profiles of nucleotide G (Figure S1 in Additional file 1). The conversions (AAG > AAA), (TAG>TAA), (CCG > CCC), (TCG > TCC) and (TTG > TTT) exhibit much higher frequency than other substitutions. Moreover, (CCG > CCC) and (TTG > TTT) are observed as the most significant patterns, followed by (AAG > AAA) and (TCG > TCC). Further investigation of the substitution patterns presented in nucleotide T shows (XCT > XCC) and (XGT > XGG) are the most frequent substitutions (Figure S2 in Additional file 1). There is also a tendency of (AAT > AAA) when the relative base position is larger than 0.5. By comparison, the nucleotide C shows distinct error patterns of (XAC > XAA), (XCC > XCA), (TTC > TTT), (TGC > TGG) and (GGC > GGG) as illustrated in Figure S3 (Additional file 1).

Next, we measure the base substitution probabilities on samples SRR1802839, SRR5685282 and ERR2180233, and the results show different patterns. Significant substitutions such as (CCA > CCC), (GGA > GGG), (AAC > AAA), (CCG > CCC), (TCG > TCC), (TTG > TTT), (CCT > CCC) and (GCT > GCC) are observed in SRR1802839, and happen at much higher rates than other conversions. For SRR5685282, (CAA > CAC), (XCA > XCC), (CGA > CGC) and (TTG > TTT) are frequently observed. Other conversions are found to have approximately same occurrence frequency. Note that the rate of errors occurring in sequences AGT, CGT and GGT is much higher than that of sequence TGT, which implies the presence of A, C and G nucleotides preceding GT will intensively increase the error rate. For ERR2180233, the substitution patterns are (XAT > XAG), (XTT > XTG), (XGT > XGG), (XAG > XAT), (XTG > XTT), (TGG > TGT) and (GGG > GGT).

We further investigate whether similar base substitution patterns can be inferred from different samples derived from same sequencing instrument. Given a base substitution type, we measure the frequency of the substitution in each base position, then use Jensen-Shannon Divergence (JSD) [30] to evaluate the similarity between inferred probability distributions. The smaller the JSD value, the more significant the result. The statistics of the JSD values of each base substitution are calculated and the results are shown in Figure S4. The median JSD of each base substitution is lower than 0.03, indicating the similar profiles are shared across different samples generated from same sequencing instrument. The dominant base substitutions in different sequencing platforms are summarized in Table S2 (Additional file 1).

The presented results demonstrate the error rates of different substitutions are jointly influenced by positional and genomic contextual information, and the profiles are different across distinct Illumina sequencing platforms, which strengthens our knowledge about the underlying patterns of base substitutions.

### Base quality distributions

**Fig. 3:**
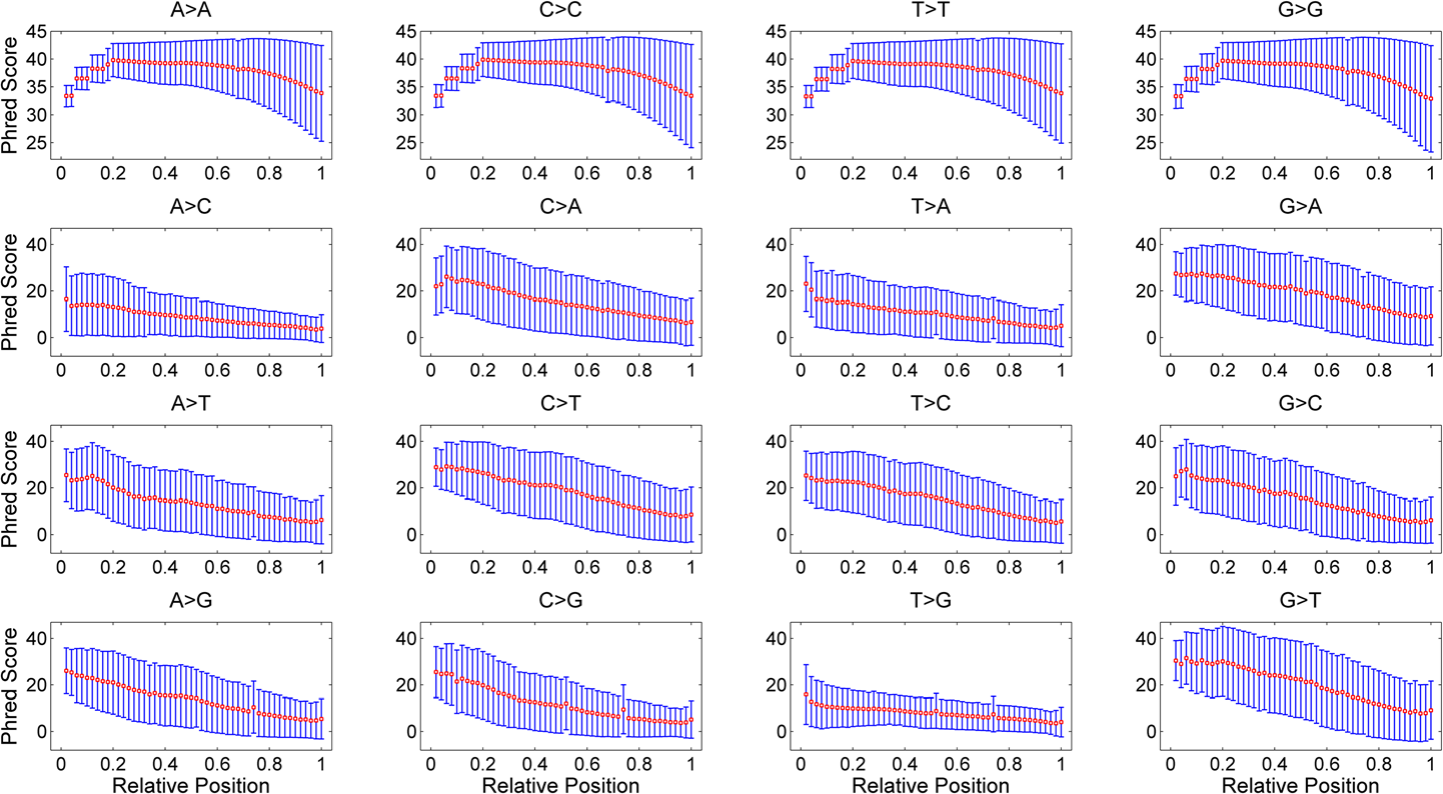
The distributions of Phred quality scores on sample SRR1614306. Base positions of each read are divided into equal-sized bins, for each of which the mean value and standard deviation of quality scores are calculated. Two nucleotides above each subplot denote the true and called bases respectively, and the relative position is calculated as the ratio between bin index and the number of bins

We evaluate the distributions of Phred quality scores on sample SRR1614306 and the results are presented in Fig. 3. For correctly called bases (A > A, C > C, T > T and G > G), the quality scores show relatively lower values near the start of the reads, and the mean values decrease from the maximum value of 40 to the minimum value of 33 towards to the end of the reads. Moreover, the per-position variance of the quality scores increases with the base position. For the erroneously called bases, similar statistical behaviors are observed for quality scores, while the mean values range from 30 to 4 when the base position increases. Analysis of SRR1802839 shows bases near two ends of the reads have lower quality values than other positions in correct calls, meanwhile the mean quality value decreases with the position if bases are erroneously called. Similar results are observed for SRR5685282 (Figure S5 in Additional file 1) except that base pair (A > C) shows remarkably lower variance of the quality scores. A much distinct profile of the quality scores is observed in ERR2180233 as shown in Figure S6 (Additional file 1). The variance of the quality scores near the start of the reads is much higher than that of other base positions.

We further evaluate the difference in mean quality scores of each base pair between sequencing platforms using Student’s t-test, and the *p*-values in Table S3 (Additional file 1) demonstrate there are significantly statistical divergences in quality scores for most of the base pairs. To examine the similarity of the base quality distributions inferred from different samples that are generated from same sequencing instrument, the JSD value of the per-position quality distribution associated with each base pair is calculated and statistically analyzed. The results in Figure S7 (Additional file 1) suggest that both Illumina Genome Analyzer IIx and HiSeq X 10 instruments show similar profiles across different samples. By comparison, much higher divergence is observed in HiSeq 2000 and HiSeq 2500 platforms, indicating the statistical behavior of base quality in these instruments may be sample-dependent.

Taken together, the presented results underscore the necessity of explicitly integrating the positional and contextual dependency of the quality scores into the simulation framework. In addition, the inferred statistics can be employed to improve discriminability between bases and improve the accuracy of read alignment tools or error correction methods [31].

### Indel distribution

To assess the difference in indel error distributions between different Illumina sequencing platforms, we infer the per-base insertion and deletion error rates and the distributions of indel lengths from the investigated samples, and the results are shown in Figures S8, S9 and S10. The insertion rate changes from 0.012 to 0.065% and the deletion rate ranges from 0.031 to 0.066%. In addition, the insertion rate is lower than deletion rate in Genome Analyzer IIx, HiSeq 2000 and HiSeq 2500 platforms. The frequency of both insertions and deletions decreases with the lengths of indels, and the dominant indels are single nucleotide insertion and deletion.

### GC-content bias

To profile the GC-content bias, we employ a locally weighted linear regression of the read counts over GC percentage, and the results are depicted in Figure S11 (Additional file 1). For all investigated samples, significant divergence is observed in the read counts values corresponding to different GC percentages. The unimodal distribution of the read counts shows the median GC percentages generally yield higher read counts, which is concordant with the previously reported results [24, 32]. Similar GC bias is observed for the samples generated from same instrument. In addition, the GC-content biases on samples ERR2180233 and ERR2180232 show a markedly different distributions when compared to other samples, which may be related to the specific technical features of HiSeq X 10 instruments.

### Simulation results

We emulate massive NGS data by introducing various genomic variations to investigate the effectiveness of the proposed method. All the simulated samples are generated by sampling reads from the chromosome 20 of hg19 human reference genome based on the sequencing profile inferred from sample SRR5685282. The produced reads are aligned to the reference genome using BWA tool, and BAM files are prepared for further analysis.

#### Consistency of the profiles

**Fig. 4:**
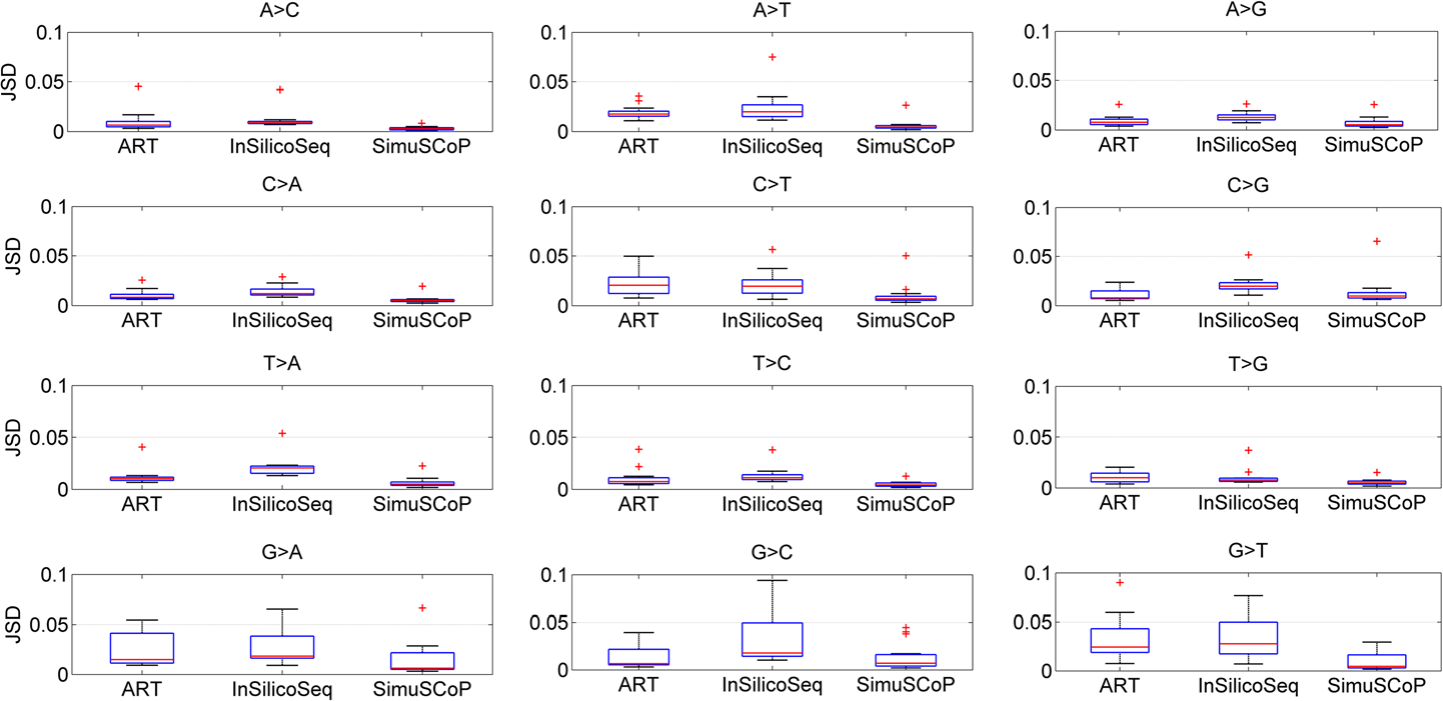
The statistics of JSD values of each base substitution. Given a base substitution type, the frequency of the substitution in each base position is measured, and Jensen-Shannon Divergence is used to evaluate the similarity between the real and inferred probability distributions. The statistics of JSD values for a given substitution are obtained by analyzing all 3-mer forms of the substitution. For instance, substitution types such as (AAA > AAC), (ACA > ACC) and (TTA > TTC) are the 3-mer forms of base substitution A > C

To examine the effectiveness of simulated data, the profiles of emulated samples generated by ART, InSilicoSeq and SimuSCoP are analyzed and compared to the ground truth values. Bowtie [33] is used to align real sequencing data when inferring the sequencing profile of InSilicoSeq as documented, and the sequencing profiles used by ART and InSilicoSeq are learned from the same real sample SRR5685282. Each method is run to generate 2 million reads. The statistics of the JSD values of each base substitution are calculated and the results are shown in Fig. 4. The median JSD values of ART and InSilicoSeq are lower than 0.05 for all base substitutions, indicating a good concordance between the inferred and real profiles. Compared to other methods, SimuSCoP gets more consistent results with the maximum median JSD value of 0.01.

**Fig. 5:**
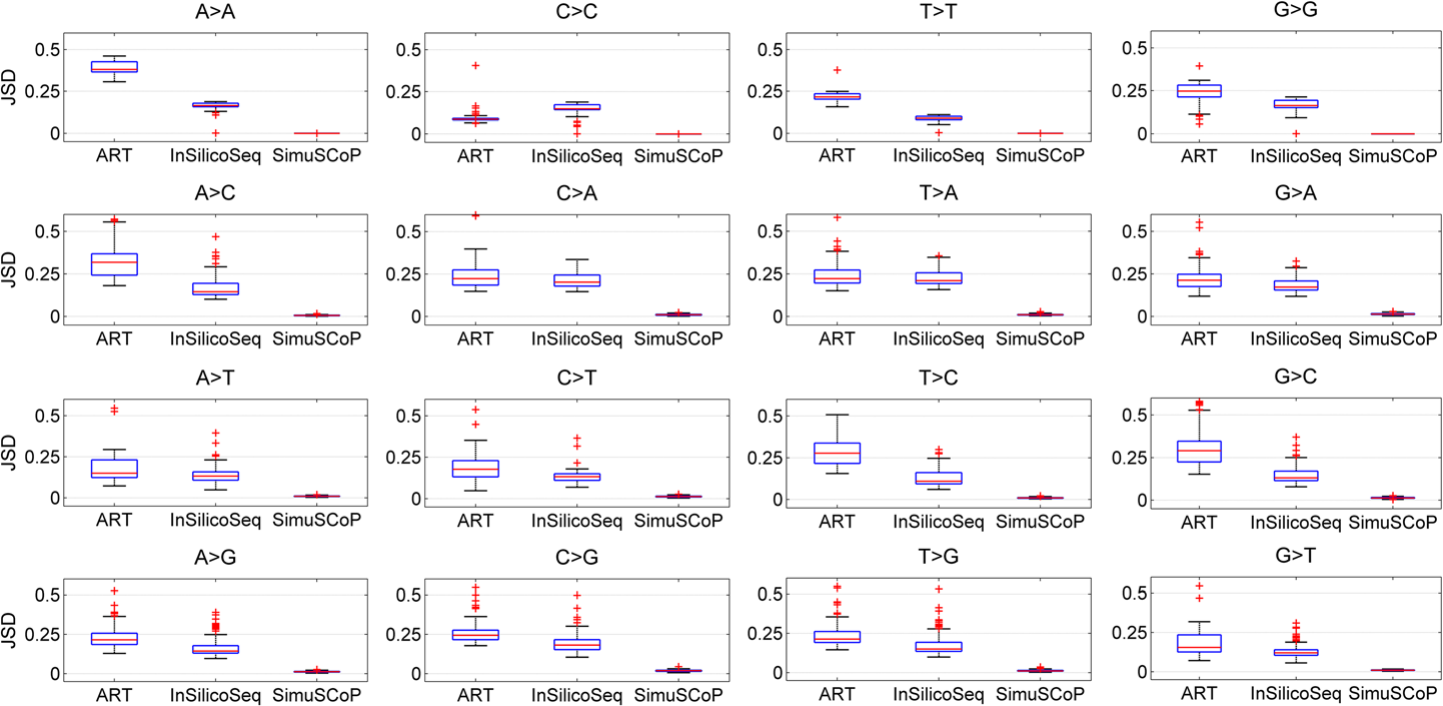
The statistics of JSD values of quality scores. Given a base pair, the JSD value of the per-position quality distribution is evaluated and statistically analyzed for each investigated simulator. Two nucleotides above each subplot denote the true and called bases respectively

We then evaluate the consistency of Phred quality scores between the simulated and real sequencing data. Figure S12 in Additional file 1 shows the mean and variance of the quality scores with respect to base positions in forward reads. All methods get very close distribution to the real data, presenting similar mean and variance values. The results demonstrate the proposed method is highly effective in revealing positional difference of quality scores. We further explore the ability of different methods in simulating contextual difference of quality scores (Fig. 5). For different contextual information, the JSD value of the per-position quality distribution is calculated and statistically analyzed to examine the consistency. ART shows much better performance in generating quality values associated with base pair (C > C) than that of other base pairs. Similarly, the performance of InSilicoSeq is also degraded in yielding quality values for most of the base pairs. By comparison, SimuSCoP maintains high significant JSD values across different base pairs, highlighting its ability of capturing both positional and contextual difference of the quality values.

These results demonstrate the proposed simulation framework is effective to generate sequencing data closely concordant with the real profiles.

#### Indel and SNV simulation

To examine the indel and SNV simulation results, 20 indels (10 insertions and 10 deletions) and 20 SNVs (10 homozygous mutations and 10 heterozygous mutations) are inserted into the chromosome 20 of the reference genome. The genome is sequenced to ~30X coverage, and the generated BAM files are analyzed using GATK Mutect2 to call somatic variations. The arguments “--genotyping-mode DISCOVERY, --output-mode EMIT_VARIANTS_ONLY, --sample-ploidy 2” are adopted for GATK Mutect2. The inferred variations are further filtered with options “DP > 20, TLOD> 15”, and the results show all indels and SNVs are correctly identified (Table S4 in Additional file 1).

#### CNV simulation

We further evaluate the CNV simulation performance, and 5 CNVs are inserted into the chromosome 20. The simulated CNVs range from one to six copies, and the size ranges from 0.5 Mb to 2 Mb. The generated sequencing data is analyzed by Control-FREEC [34] using the arguments “ploidy=2, window=1000, step=1000”. A CNV is considered to be accurately called only if any predicted CNV covers the 85% size of the CNV and has equal copy number with the CNV. The results show all simulated CNVs are correctly identified (Table S5 in Additional file 1).

#### Tumor sample simulation

To assess the ability of SimuSCoP in simulating tumor samples, we generate 2 tumor clones by introducing different aberrations including LOH and heterozygous events into the normal genome as shown in Table S6 (Additional file 1), then mix the tumor and normal genomes at different proportions (Table S7 in Additional file 1). The produced sequencing data is analyzed using CLImAT-HET [35] to infer copy number alterations and clonal heterogeneity. Table S7 shows the predicted tumor purity is significantly correlated with the ground truth (correlation coefficient = 0.99, *p*-value = 5.78 × 10^− 10^). In addition, the simulated aberrations and corresponding cell fractions are accurately inferred, an example of prediction results on a simulated heterogeneous sample (50% Clone1, 40% Clone2 and 10% normal cells) is shown in Figure S13 (Additional file 1). Two clonal clusters are correctly identified with corresponding cell fractions of 0.49 and 0.86 respectively, meanwhile 15 out of 16 segments are assigned with the correct clonal cluster and tumor genotype. These results demonstrate the ability of SimuSCoP in reliably emulating complex tumor samples.

#### Runtime performance

To test the computation and memory efficiency of SimuSCoP, different volumes of data are generated under distinct computational constraints (Table 2). The evaluation is performed on a workstation with 16 GB memory and 16-core Dual Xeon E5–2620 CPU. The results show that processing time presents nearly linear reduction and memory consumption is gradually aggravated when more threads are used. For instance, using 8 threads to generate 21 million reads needs 4.62 min time and 893 MB peak memory, showing 83% time saving and nearly 3 times memory consumption increasing when compared to single thread mode. On the other hand, simulating larger volume of data does not extensively increase the required memory. For instance, generating one more 2.1 million reads just needs ~ 10 MB per-thread extra memory consumption. For comparison, we also evaluate the runtime performance of ART and InSilicoSeq by generating simulation datasets under same coverage. ART is implemented to run in single-thread mode, it uses nearly 1.1 min to generate 2.1 million reads and the used time linearly increases with sequencing coverage, while the memory consumption is about 286 MB and keeps unchanged when the sequencing coverage increases. The runtime efficiency of SimuSCoP is comparable to that of ART when using multiple threads. InSilicoSeq needs approximately 29 min to simulate 2.1 million reads under default configuration, and fails to generate larger datasets due to excessive memory usage.

**Table 2:** The runtime performance of SimuSCoP

Coverage	Number of reads (million)	Number of threads	Time (min)	Peak memory (MB)
5	2.1	1	2.76	145
		2	1.58	156
		4	0.87	179
		8	0.57	234
10	4.2	1	5.67	155
		2	3.08	176
		4	1.70	227
		8	1.03	324
20	8.4	1	11.13	177
		2	6.10	224
		4	3.35	308
		8	1.88	476
50	21.0	1	27.15	225
		2	15.12	321
		4	8.15	511
		8	4.62	893

## Discussion

As SimuSCoP can infer more accurate sequencing profiles from real datasets, it is useful for providing more accurate evaluation of the real performance of downstream variant calling tools. Table S8 (Additional file 1) shows the SNV detection sensitivity of GATK by analyzing the sequencing data generated by ART and SimuSCoP. The results imply a number of heterozygous SNVs are not called from the sequencing data generated by SimuSCoP at lower sequencing coverage, suggesting further improvement in the performance of GATK may be achieved by considering the sequencing profiles obtained by SimuSCoP. However, how to incorporate the learned profiles into the variant calling process is another interesting topic that needs to be extensively investigated, and we plan to study this potential research direction in the future.

## Conclusions

Simulation of NGS data has been a long-standing interest in the literature, and numerous bioinformatics tools have been developed for this propose. An overview of current NGS simulators is provided in this work to show the difference in functional implementations and supported applications of each tool. By making comparative analysis, we point out the functional and runtime limitations of the existing simulators, and underscore the necessity of developing new bioinformatics tools that are more effective and easy-to-use.

To overcome the downsides of current simulators, we introduce a novel simulation framework called SimuSCoP to reliably emulating complex NGS dataset. To effectively represent the read generation procedure, a probability model is employed to investigate the patterns of base substitutions and statistical differences of Phred quality scores from both positional and contextual views. Analysis of real sequencing data suggests that there are significant divergences in base substitution patterns and quality score distributions between different Illumina sequencing platforms, demonstrating the necessity of integrating such knowledge into the read simulation models. By using the inferred profiles, an integrated read simulation pipeline is implemented by incorporating the correlated biological and technological features into one framework. Finally, the evaluation of our tool from multiple aspects shows its high effectiveness, functionality and efficiency.

Taken together, we believe that the presented work will catalyse new development of downstream bioinformatics methods for analyzing NGS data.

## Availability and requirements

Project name: SimuSCoP.

Project home page: https://github.com/qasimyu/simuscop

Operating system(s): Linux system.

Programming language: C++.

Other requirements: CMake 2.8 or higher.

License: GNU General Public License V3.

Any restrictions to use by non-academics: License needed.

## Supplementary information

**Additional file 1: Figure S1.** Presentations of Supplementary Figures and Tables.

## Data Availability

The samples analyzed in this study can be downloaded from the Sequence Read Archive (SRA) of NCBI.

## Abbreviations

NGS: Next-generating sequencing
SNV: Single nucleotide variation
CNV: Copy number variation
LOH: Loss of heterozygosity
SV: Structure variations
HMM: Hidden Markov models
DOC: Depth of coverage
WGS: Whole-genome sequencing
ACN: Average copy number
