# Supplementary material for: SimuSCoP: reliably simulate Illumina sequencing data based on position and context dependent profiles
Source: BMC Bioinformatics. 2020 Jul 23;21:331. doi: 10.1186/s12859-020-03665-5 (PMC7379788; doi:10.1186/s12859-020-03665-5)
Supplement: Supplementary file 1 — Additional file 1: Figure S1. Presentations of Supplementary Figures and Tables. [file 12859_2020_3665_MOESM1_ESM.pdf]

SimuSCoP: reliably Simulate Illumina Sequencing data based on position and Context dependent Profiles

Supplementary Material

Zhenhua Yu, Fang Du, Rongjun Ban and Yuanwei Zhang

1. Supplementary Figures

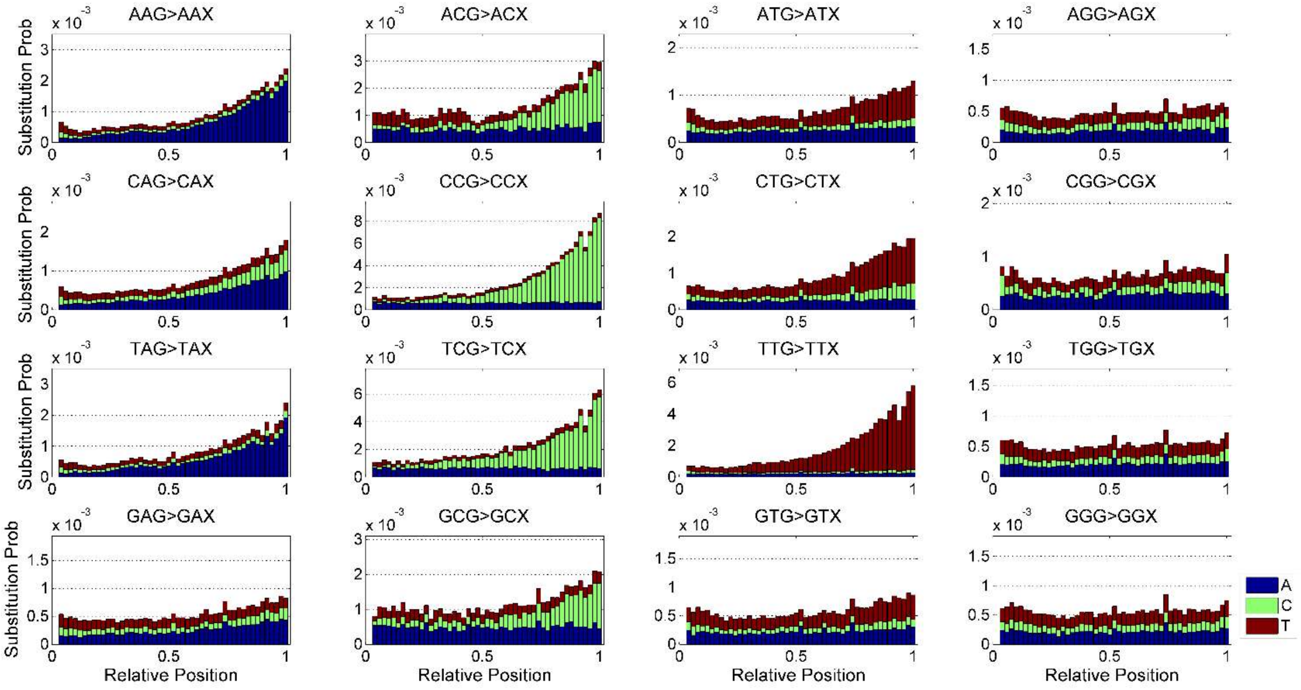

Figure S1. The probabilities of substituting nucleotide G for other nucleotides in forward reads of sample SRR1614306. The conditional occurrence frequency of each base substitution under 3-mer bases derived from source sequence is measured.

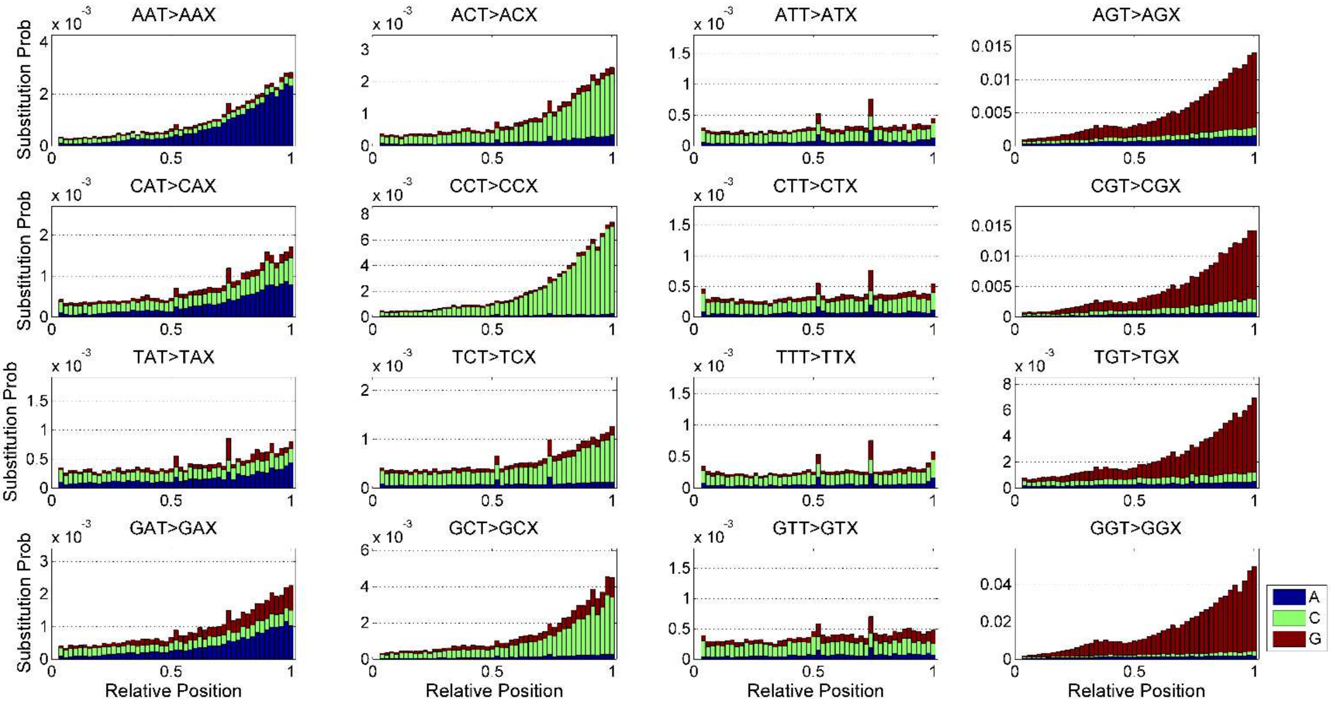

Figure S2. The probabilities of substituting nucleotide T for other nucleotides in forward reads of sample SRR1614306.

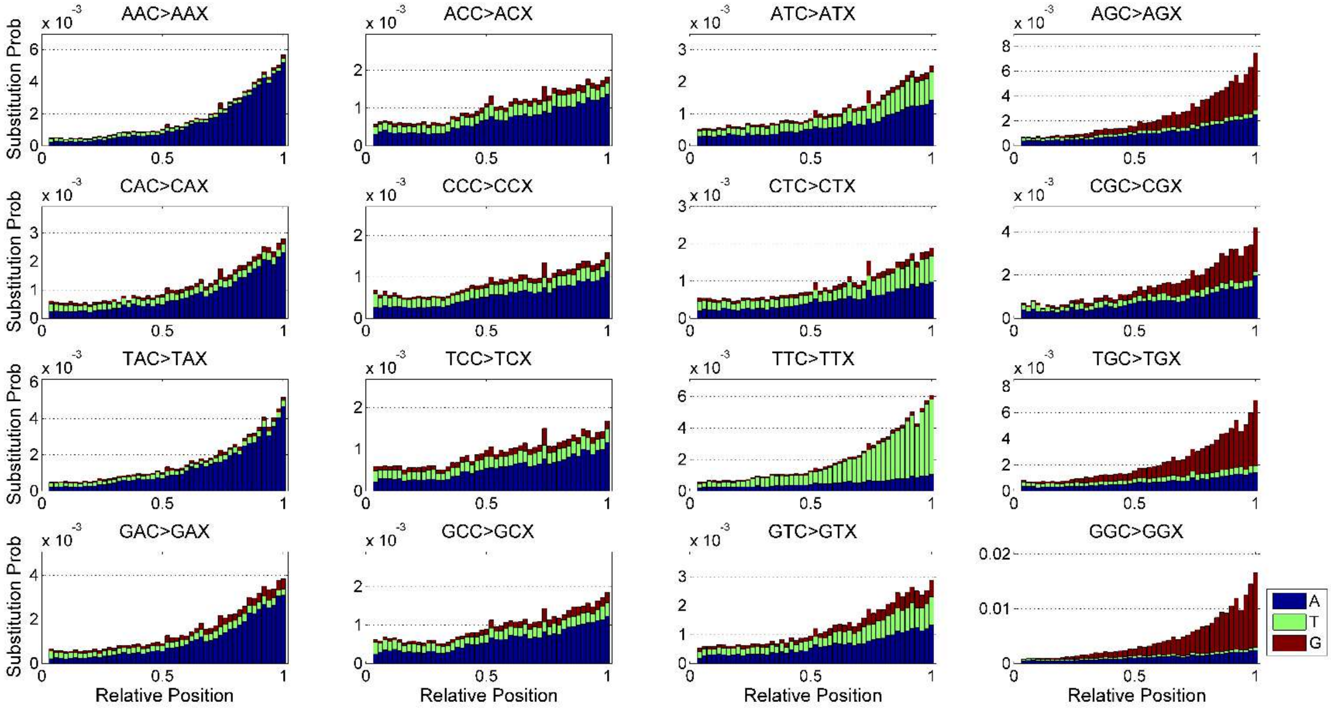

Figure S3. The probabilities of substituting nucleotide C for other nucleotides in forward reads of sample SRR1614306.

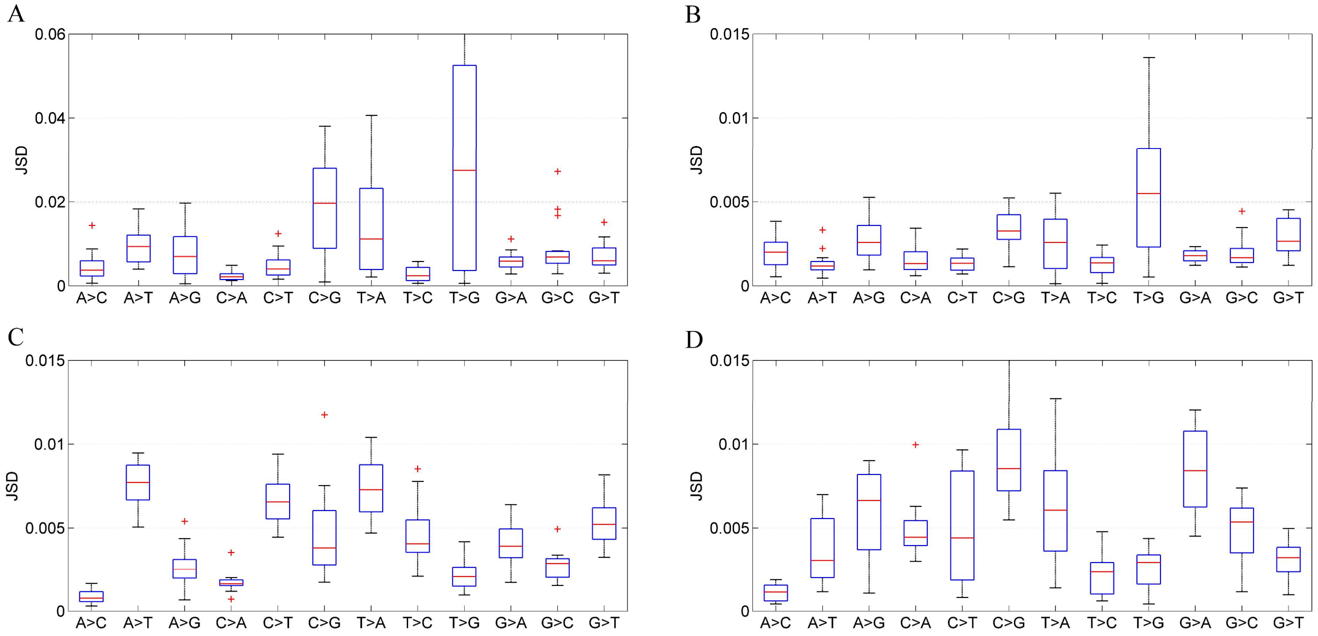

Figure S4. Comparison of the similarity between base substitution profiles inferred from same sequencing instrument. The results are inferred for Illumina Genome Analyzer IIx (**A**), HiSeq 2000 (**B**), HiSeq 2500 (**C**) and HiSeq X Ten (**D**) instruments respectively. The statistics of JSD values for a given substitution are obtained by analyzing all 3-mer forms of the substitution. For instance, substitution types such as (AAA>AAC), (ACA>ACC) and (TTA>TTC) are the 3-mer forms of base substitution A>C.

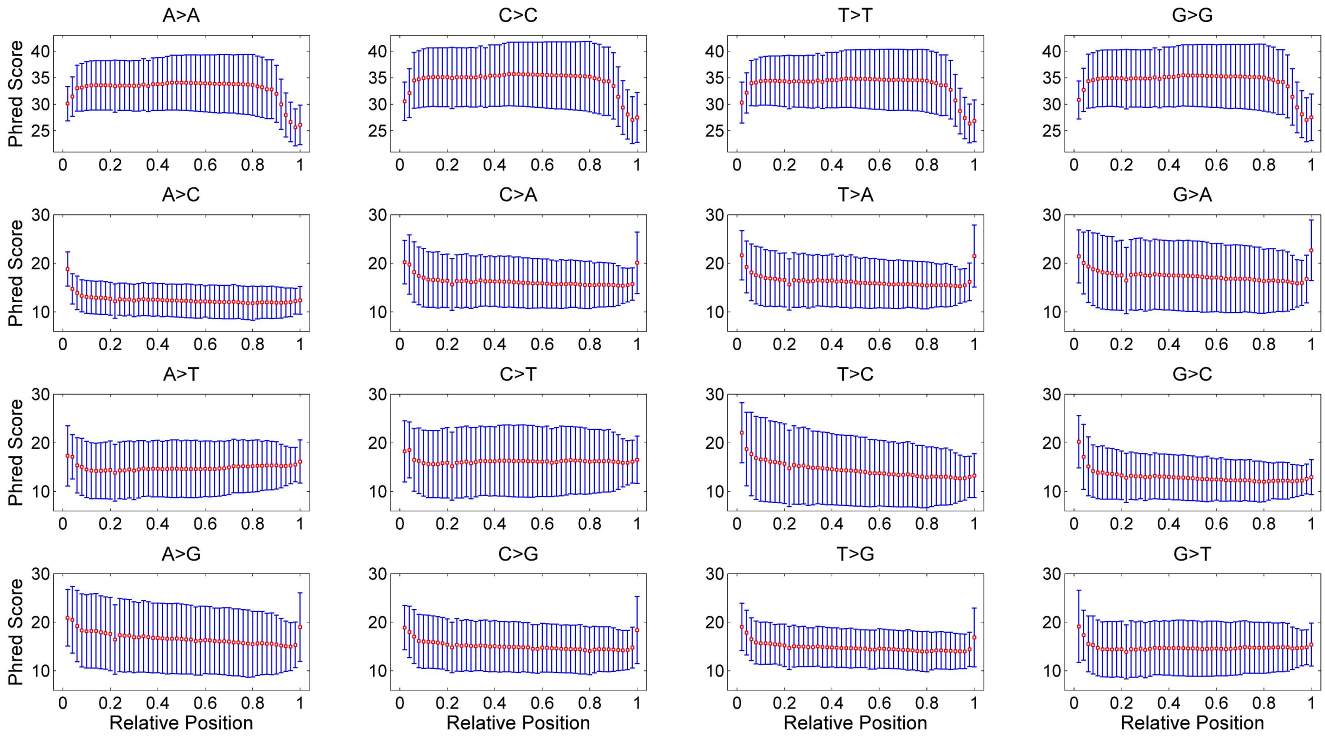

Figure S5. The distributions of Phred quality scores on sample SRR5685282. Base positions of each read are divided into equal-sized bins, for each of which the mean value and standard deviation of quality scores are calculated. Two nucleotides above each subplot denote the true and called bases respectively, and the relative position is calculated as the ratio between bin index and the number of bins.

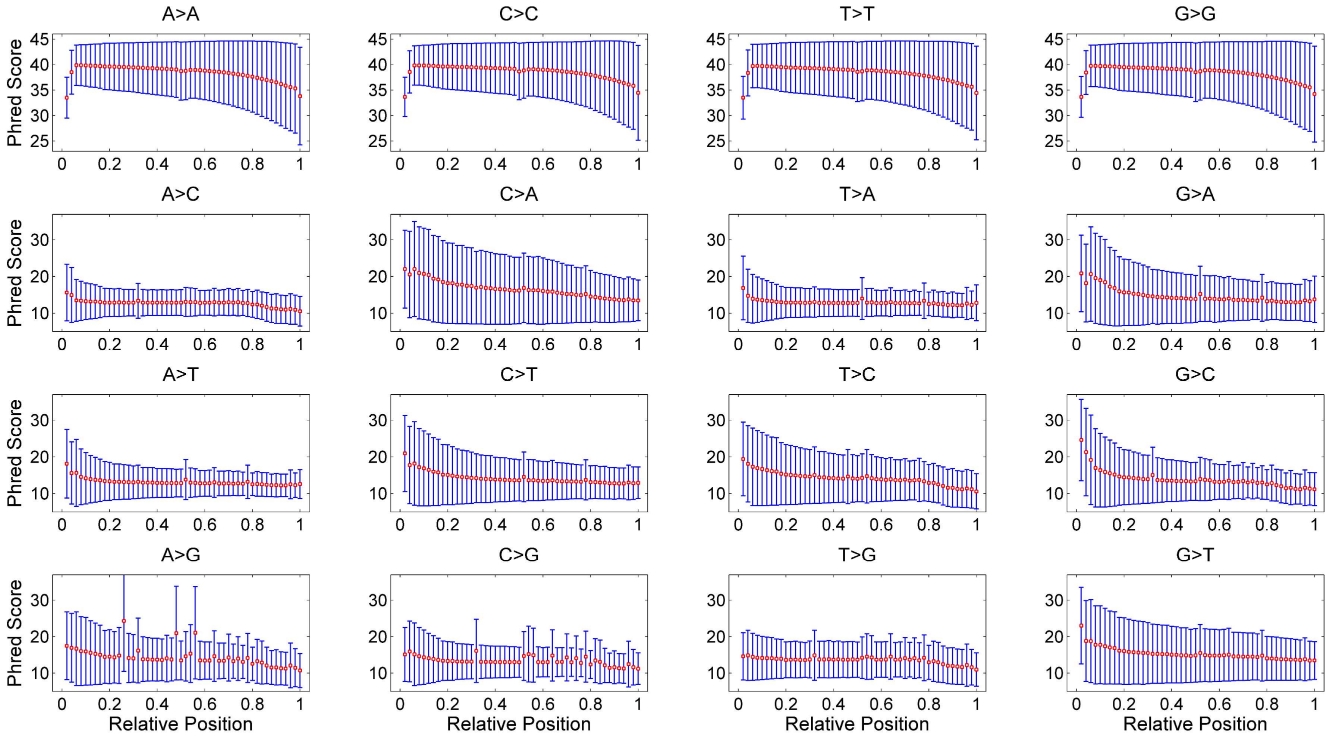

Figure S6. The distributions of Phred quality scores on sample ERR2180233.

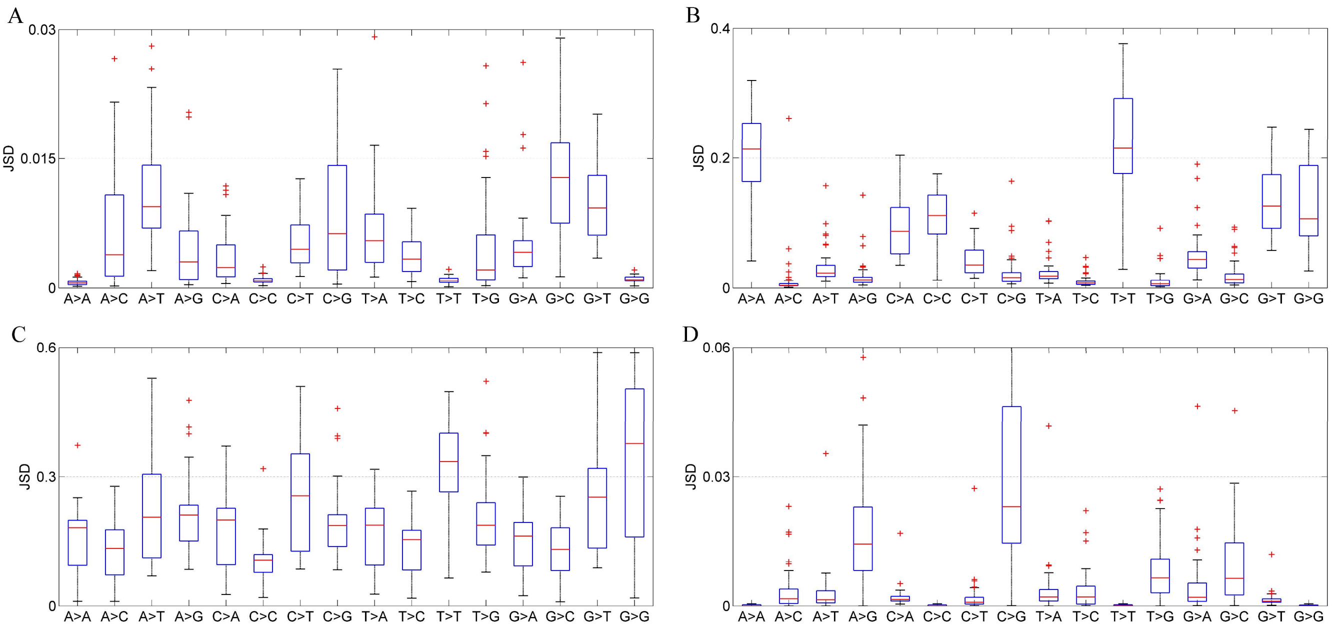

Figure S7. Comparison of the similarity between base quality profiles inferred from same sequencing instrument. The results are inferred for Illumina Genome Analyzer IIx (**A**), HiSeq 2000 (**B**), HiSeq 2500 (**C**) and HiSeq X Ten (**D**) instruments respectively. The JSD value of the per-position quality distribution associated with each base pair is calculated and statistically analyzed.

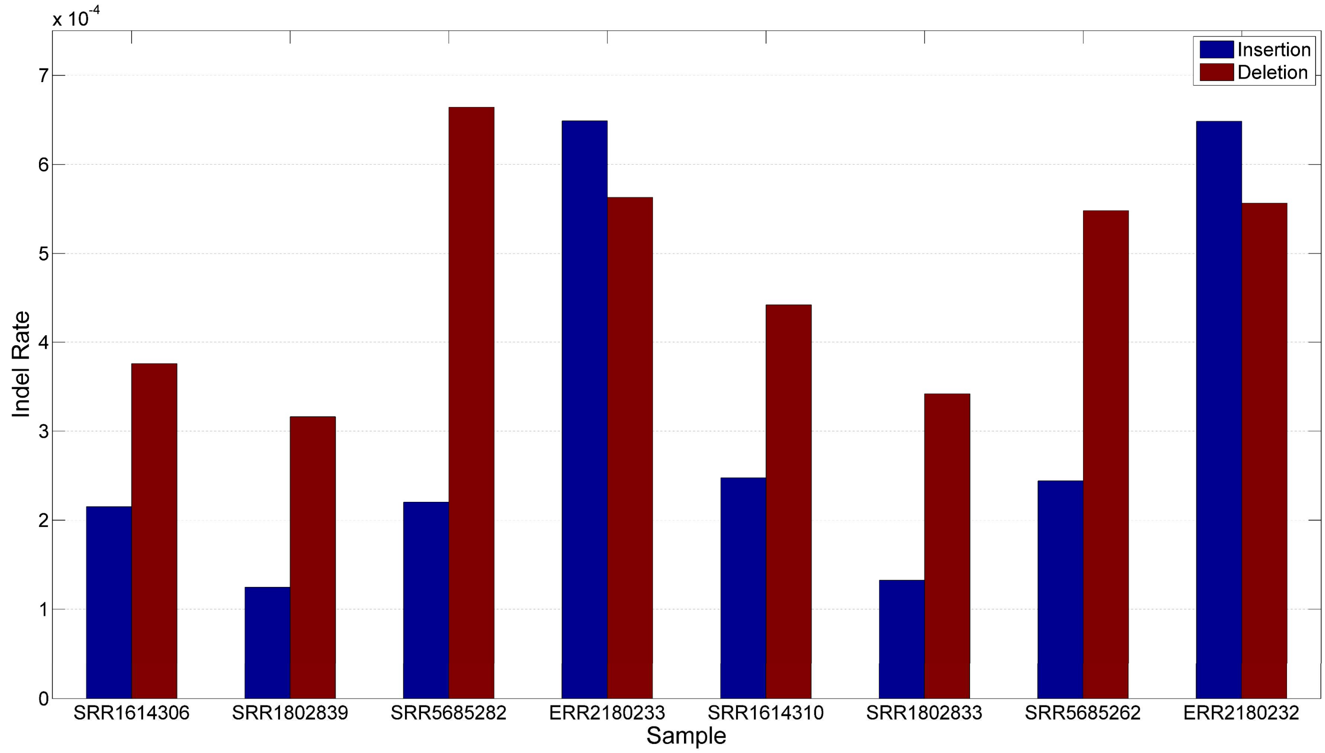

Figure S8. Insertion and deletion error rate inferred from real samples.

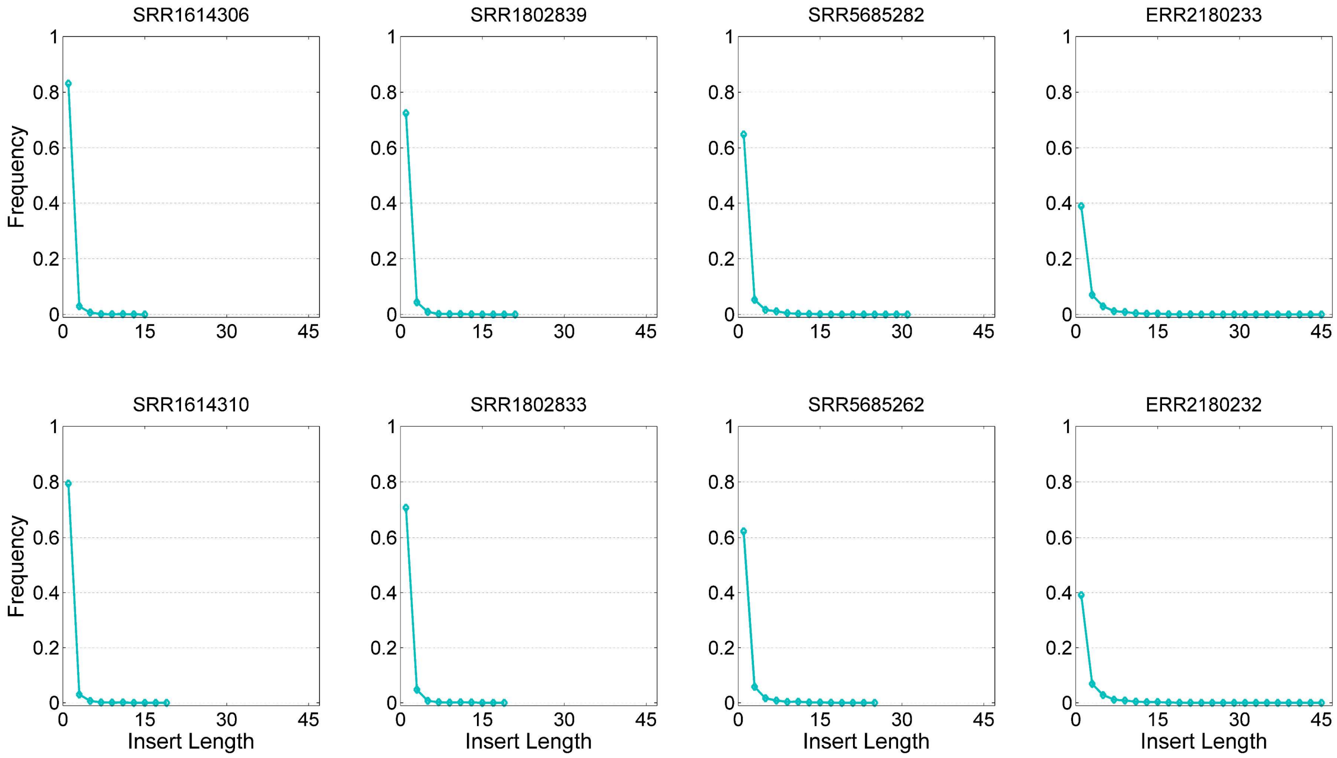

Figure S9. The distribution of insertion length inferred from real samples.

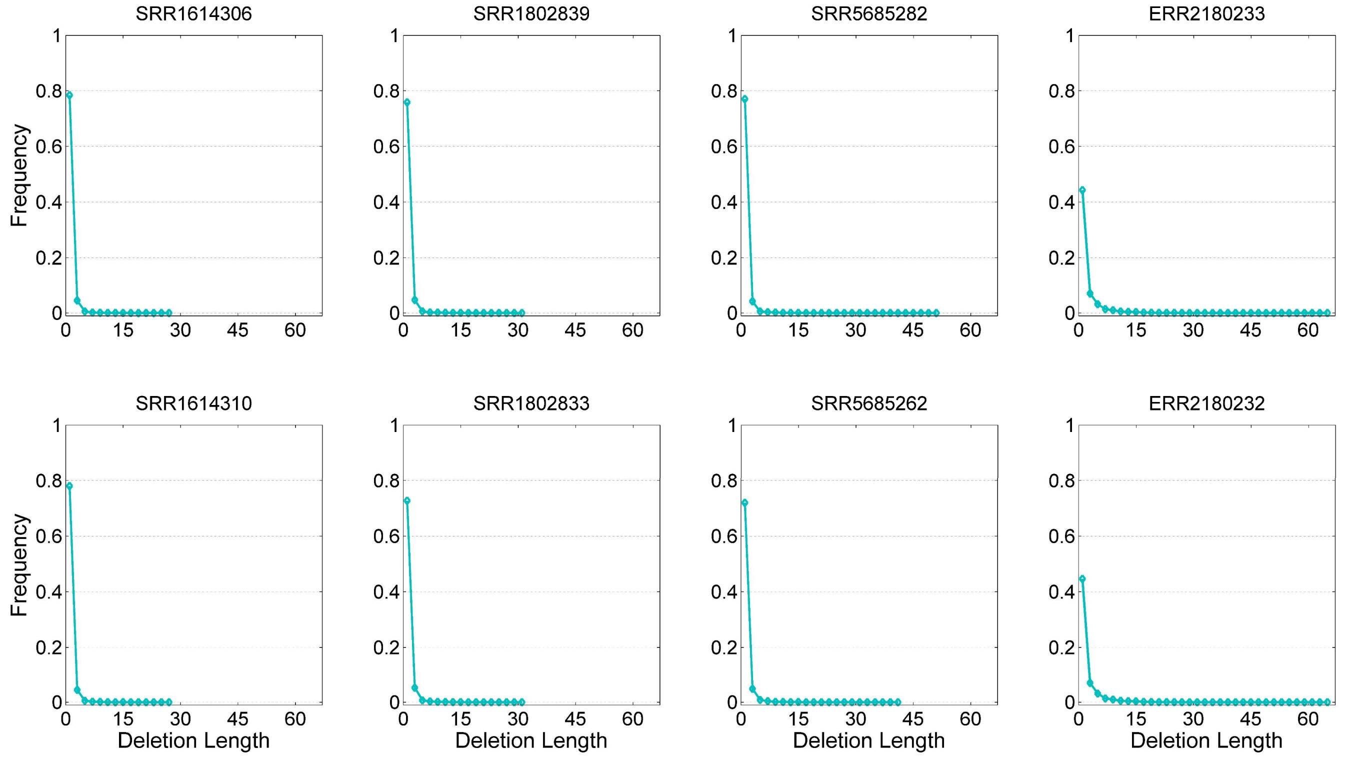

Figure S10. The distribution of deletion length inferred from real samples.

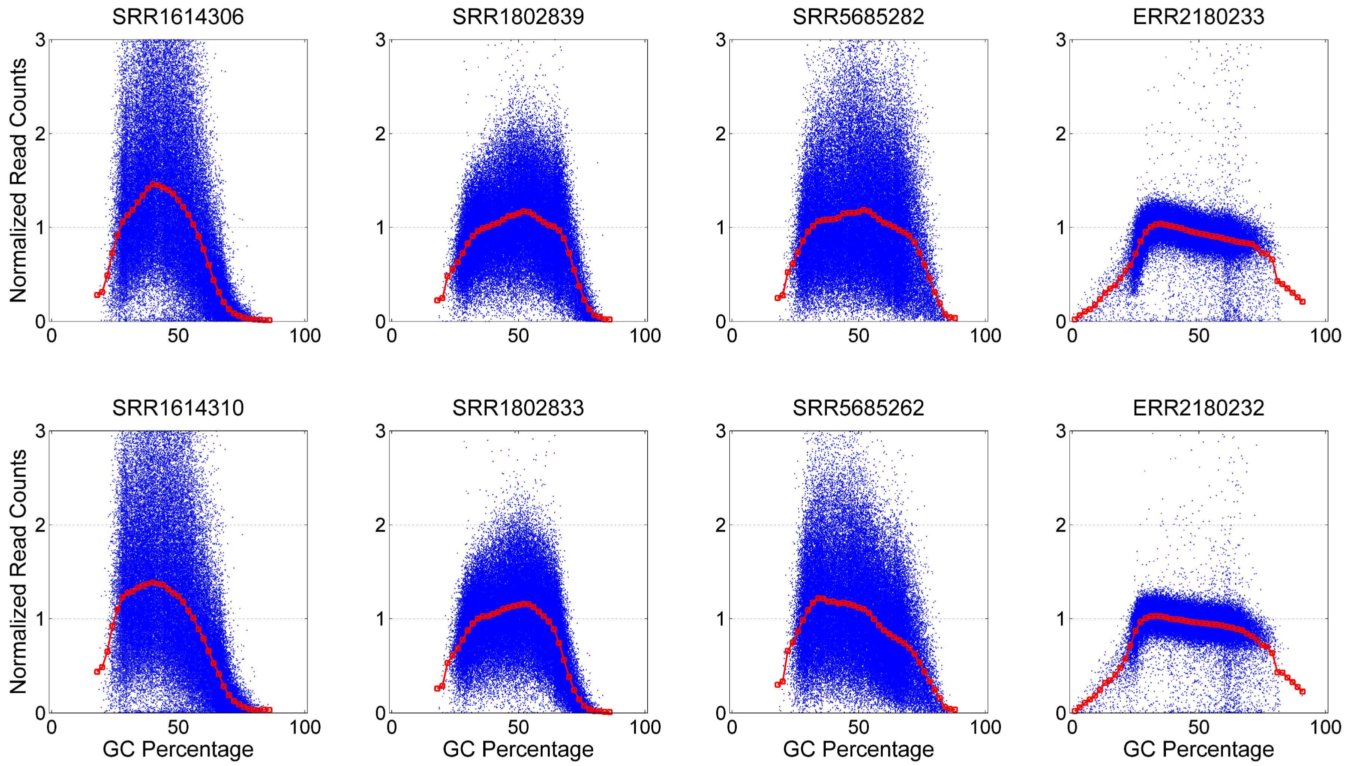

Figure S11. The distribution of normalized read counts over GC-content inferred from different samples. Locally weighted linear regression is used to model the relationship between read counts and GC-content.

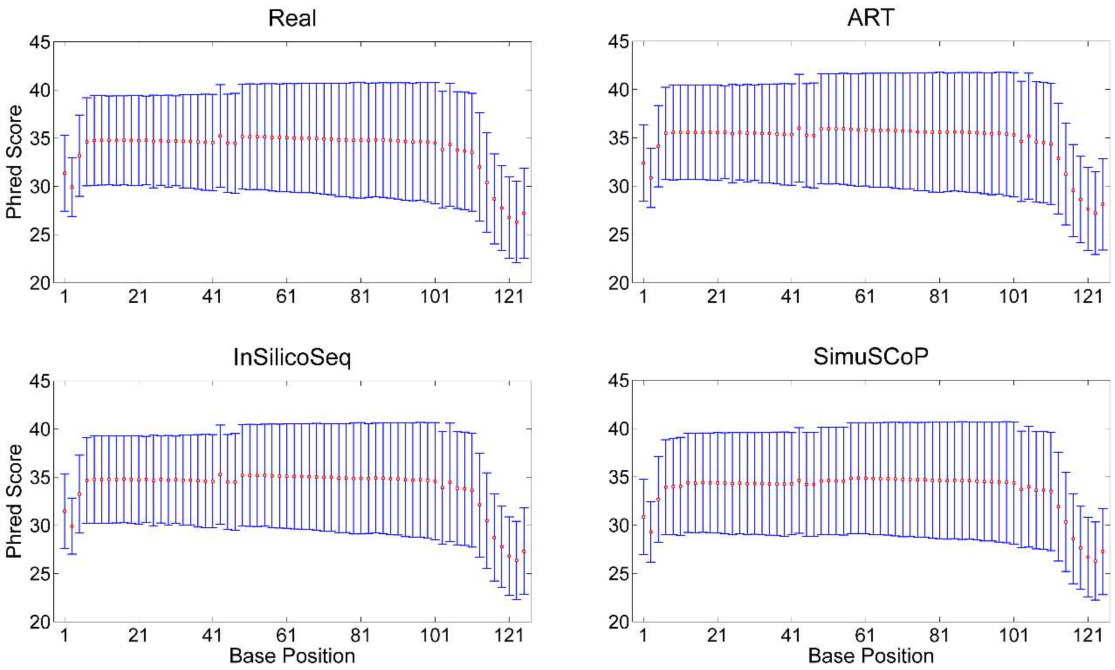

Figure S12. The per-position distribution of quality scores in forward simulated and real reads. The mean and variance of the quality scores corresponding to each base position are measured. All investigated simulators get close distribution to the real data.

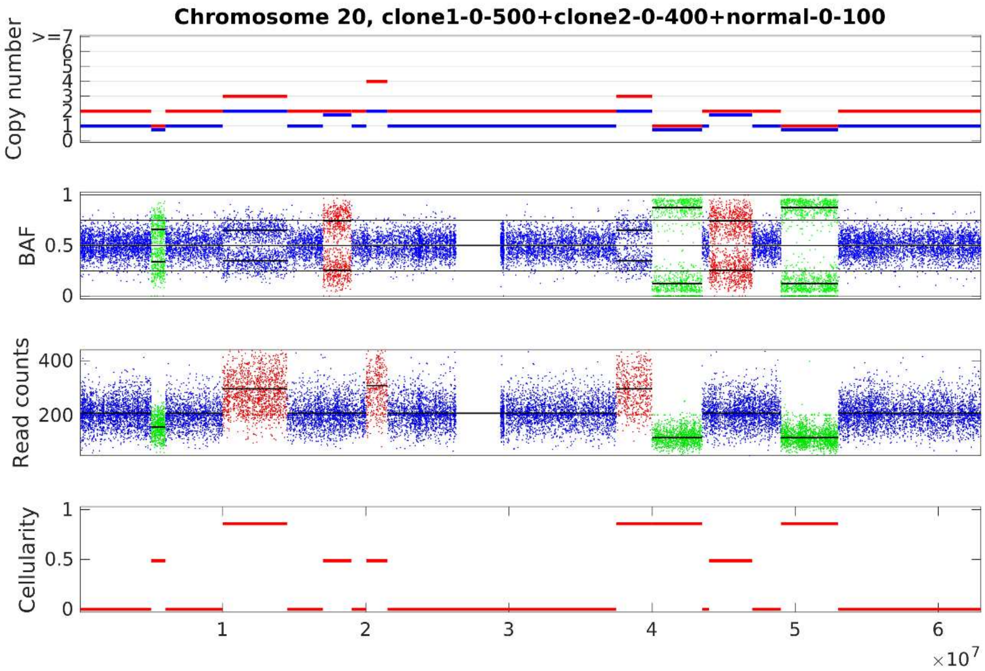

Figure S13. The aberration detection results on a simulated heterogeneous tumor sample. The sample is analyzed using CLImAT-HET software, and two clonal clusters are correctly identified with corresponding cell fractions of 0.49 and 0.86 respectively, meanwhile 15 out of 16 segments are assigned with the correct clonal cluster and tumor genotype.

2. Supplementary Tables

Table S1. The accession Ids of the samples downloaded from SRA.

Sequencing instrument	Ids of samples
Genome Analyzer IIx	SRR1614306, SRR1614310
HiSeq 2000	SRR1802839, SRR1802833
HiSeq 2500	SRR5685282, SRR5685262
HiSeq X Ten	ERR2180233, ERR2180232

Table S2. The dominant base substitutions in different sequencing platforms inferred from real datasets.

Sequencing platform	Dominant base substitutions
Genome Analyzer IIx	(XCA>XCC), (TTA>TTT), (TGA>TGG), (GGA>GGG), (AAG>AAA), (TAG>TAA), (CCG>CCC), (TCG>TCC), (TTG>TTT), (XCT>XCC),(XGT>XGG), (XAC>XAA), (XCC>XCA), (TTC>TTT), (TGC>TGG), (GGC>GGG)
HiSeq 2000	(CCA>CCC), (GGA>GGG), (AAC>AAA), (CCG>CCC), (TCG>TCC), (TTG>TTT), (CCT>CCC), (GCT>GCC)
HiSeq 2500	(CAA>CAC), (XCA>XCC), (CGA>CGC), (TTG>TTT)
HiSeq X Ten	(XAT>XAG), (XTT>XTG), (XGT>XGG), (XAG>XAT), (XTG>XTT), (TGG>TGT), (GGG>GGT)

Table S3. The p-values of Student's t-tests. T-test is adopted to evaluate the difference in mean quality scores of each base pair between sequencing platforms.

Base pairs	Sequencing platforms comparison*					
	GA v. 2000	GA v. 2500	GA v. XTen	2000 v. 2500	2000 v. XTen	2500 v. XTen
A>A	6.78e-33	2.30e-31	2.49e-03	7.96e-02	5.76e-35	1.81e-33
A>C	2.26e-09	2.20e-01	2.58e-02	7.18e-17	1.87e-31	2.97e-01
A>T	2.65e-08	3.17e-15	2.66e-05	3.53e-42	1.89e-33	9.22e-31
A>G	4.90e-17	4.98e-02	1.01e-07	3.40e-29	2.94e-27	1.18e-16
C>A	1.51e-14	2.07e-11	3.14e-12	1.86e-38	1.89e-38	1.07e-01
C>C	7.69e-24	6.75e-25	7.38e-05	4.16e-01	8.77e-34	5.48e-27
C>T	3.05e-15	3.15e-01	1.08e-01	1.00e-45	1.38e-47	8.09e-04
C>G	1.92e-16	1.01e-02	6.60e-02	3.00e-15	6.03e-26	4.52e-06
T>A	7.83e-07	2.00e-01	4.27e-01	5.11e-09	2.02e-05	7.65e-18
T>C	3.61e-15	1.60e-02	1.50e-06	1.24e-08	1.94e-05	1.42e-12
T>T	5.70e-33	1.59e-27	5.06e-04	1.16e-01	3.49e-36	5.27e-30
T>G	7.44e-09	7.78e-06	2.97e-06	3.73e-02	9.13e-02	6.32e-03
G>A	2.10e-08	2.81e-04	4.45e-01	8.63e-33	5.97e-17	1.85e-09
G>C	7.56e-08	5.55e-04	1.13e-01	9.37e-31	1.76e-25	1.64e-13
G>T	5.63e-19	8.17e-30	5.81e-25	1.14e-52	3.40e-47	3.18e-11
G>G	3.38e-21	2.81e-24	1.27e-05	4.67e-01	2.68e-31	6.77e-28

*: GA, 2000, 2500 and XTen denote Illumina Genome Analyzer IIx, HiSeq 2000, HiSeq 2500 and HiSeq X Ten instruments, respectively.

Table S4. The detailed information of simulated indels and SNVs. All variations are accurately called by GATK Mutect2.

CHROM	POS	REF	ALT	DP	TLOD
20	1300099	C	CTCGAGTCGAG	54	150.88
20	2000100	A	T	73	130.15
20	2500099	T	TTCGAGTCGA	55	141.33
20	3000099	AGATGCTCCTC	A	105	241.28
20	4000100	T	G	92	174.84
20	4500099	T	TTCGAGTCG	38	60.66
20	4600100	T	C	49	38.11
20	5000099	TGACCAAGGG	T	122	239.92
20	7000100	T	G	131	145.63
20	7500100	A	T	56	26.98
20	8000100	T	G	118	156.39
20	8500100	A	G	28	15.59
20	9000100	G	C	102	185.88
20	9500099	AGATAAATT	A	50	142.48
20	11000099	G	GTCGAGTC	97	249.27
20	11500100	G	C	28	43.95
20	12000099	G	GTCGAGT	84	127.82
20	13000100	T	C	93	84.11
20	13500099	CAAGGCTG	C	79	222.12
20	14000099	TTAGTAG	T	112	225.50
20	37000096	AAAAGGTCTCG	A	87	164.94
20	39000100	T	G	116	103.26
20	40000100	G	C	97	85.24
20	41000099	C	CTCGAGTCGAG	161	316.58
20	42000100	A	C	138	143.65
20	43000098	T	TATCGAGTCG	123	277.16
20	43500100	A	T	45	52.52
20	44000099	T	TTCGAGTCG	105	259.17
20	46000100	C	T	115	118.50
20	47000099	CTGATTATGA	C	128	318.03
20	48000099	AAACCAGGG	A	104	235.58
20	51000100	G	A	104	60.39
20	53000100	C	G	113	112.28
20	55000100	C	T	102	161.95
20	56000100	A	T	103	204.92
20	57000099	T	TTCGAGTC	119	225.58
20	58000099	AGCCCGAG	A	113	187.37
20	59000100	A	T	87	122.17
20	61000099	G	GTCGAGT	94	217.57
20	62000099	GGCTTTC	G	133	281.70

Table S5. The detailed information of simulated CNVs. Sequencing data is analyzed using Control-FREEC.

**Chr**	**Start Position**	**End position**	**Copy number**	**CNV size (MB)**	**Detection result***
20	5000000	5500000	5	0.5	X
20	17000000	18500000	3	1.5	X
20	30000000	31000000	6	1.0	X
20	40000000	41800000	4	1.8	X
20	49000000	51000000	1	2.0	X

*: X indicates the given CNV is accurately detected.

Table S6. The detailed information of simulated heterogeneous tumor samples.

**Aberration**					**Cell population***		
Chr	Start position	End position	Copy number	Major copy number	Clone1	Clone2	Normal
20	5000000	6000000	1	1	X		
20	10000000	14500000	3	2	X	X	
20	17000000	19000000	2	2	X		
20	20000000	21500000	4	2	X		
20	37500000	40000000	4	3	X		
20	40000000	43500000	1	1	X	X	
20	44000000	47000000	2	2	X		
20	49000000	53000000	1	1	X	X	

*: X indicates a given aberration is carried by a cell population.

Table S7. The mixed and predicted proportions of different cell populations.

**Mixed proportions**			**Predicted proportions**		
Clone1	Clone2	Normal	Clone1	Clone2	Normal
0.20	0	0.80	0.23	0	0.77
0.40	0	0.60	0.40	0	0.60
0.60	0	0.40	0.58	0	0.42
0.80	0	0.20	0.77	0	0.23
1.00	0	0	0.99	0	0.01
0.35	0.35	0.30	0.36	0.30	0.34
0.35	0.45	0.20	0.35	0.41	0.24
0.50	0.40	0.10	0.49	0.37	0.14
0.70	0.30	0	0.67	0.32	0.01

Table S8. Analysis of the effects of the sequencing profiles inferred by SimuSCoP on SNV detection sensitivity.

**Sequencing coverage**	**SNV detection sensitivity***	
	Homozygous SNVs	Heterozygous SNVs
10	1.00/1.00	0.99/0.93
20	1.00/1.00	0.99/0.97
30	1.00/1.00	0.99/0.99

*: The data is presented as ART/SimuSCoP. A 1MB-length sequence sampled from chromosome 20 of hg19 is used as the reference sequence, and further fine-tuned by randomly inserting 1000 SNVs. ART and SimuSCoP are run to yield sequencing data based on the sequencing profiles inferred from the real sample SRR5685282. The generated reads are aligned to reference using BWA tool, and SNVs are detected using GATK HaplotypeCaller under default parameters. Sensitivity is defined as the ratio of the number of accurately detected SNVs and the number of all SNVs.
